# Boron-Based Inverse Sandwich V_2_B_7_^−^ Cluster: Double π/σ Aromaticity, Metal–Metal Bonding, and Chemical Analogy to Planar Hypercoordinate Molecular Wheels

**DOI:** 10.3390/molecules28124721

**Published:** 2023-06-12

**Authors:** Peng-Fei Han, Qiang Sun, Hua-Jin Zhai

**Affiliations:** 1 Nanocluster Laboratory, Institute of Molecular Science, Shanxi University, Taiyuan 030006, China; 201722801006@email.sxu.edu.cn; 2 Center for Applied Physics and Technology, School of Materials Science and Engineering, Peking University, Beijing 100871, China; sunqiang@pku.edu.cn

**Keywords:** boron-based inverse sandwich clusters, double π/σ aromaticity, metal–metal bonding, electronic transmutation, chemical bonding

## Abstract

Inverse sandwich clusters composed of a monocyclic boron ring and two capping transition metal atoms are interesting alloy cluster systems, yet their chemical bonding nature has not been sufficiently elucidated to date. We report herein on the theoretical prediction of a new example of boron-based inverse sandwich alloy clusters, V_2_B_7_^−^, through computational global-minimum structure searches and quantum chemical calculations. This alloy cluster has a heptatomic boron ring as well as a perpendicular V_2_ dimer unit that penetrates through the ring. Chemical bonding analysis suggests that the inverse sandwich cluster is governed by globally delocalized 6π and 6σ frameworks, that is, double 6π/6σ aromaticity following the (4*n* + 2) Hückel rule. The skeleton B−B σ bonding in the cluster is shown not to be strictly Lewis-type two-center two-electron (2c-2e) σ bonds. Rather, these are quasi-Lewis-type, roof-like 4c-2e V−B_2_−V σ bonds, which amount to seven in total and cover the whole surface of inverse sandwich in a truly three-dimensional manner. Theoretical evidence is revealed for a 2c-2e Lewis σ single bond within the V_2_ dimer. Direct metal–metal bonding is scarce in inverse sandwich alloy clusters. The present inverse sandwich alloy cluster also offers a new type of electronic transmutation in physical chemistry, which helps establish an intriguing chemical analogy between inverse sandwich clusters and planar hypercoordinate molecular wheels.

## 1. Introduction

As a nearest neighbor of carbon in the periodic table, boron features structural diversity and nonclassical chemical bonding, as do its chemical compounds [1]. Elemental boron clusters have been studied both experimentally and theoretically since the 1980s. Anderson and coworkers reported the mass spectra of cationic boron clusters and conducted collision-induced dissociation (CID) experiments, which led to the discovery of magic boron clusters such as B_13_^+^ [2]. Subsequently, the structural and electronic properties of boron clusters have been probed since the 2000s in an array of combined experimental and computational works by Zhai, Wang, and coworkers, in which gas-phase photoelectron spectroscopy (PES) is utilized as a primary technique. These studies showed that anionic boron clusters with up to 40 atoms (excluding B_39_^−^) assume planar or quasi-planar structures [3,4,5,6,7,8,9,10,11,12,13,14], which represent a highly unusual system in cluster science, being governed entirely by the intrinsic electron deficiency of boron. For cationic boron clusters, planar or quasi-planar structures were observed up to 15 atoms in size, according to ion mobility experiments [15]. In contrast, indirect evidence was revealed for a planar-to-tubular structure transition in neutral boron clusters at around 20 atoms, basing on a joint experimental and computational study on anionic B_20_^−^ cluster [16]. The unique geometric structures of elemental boron clusters are dictated by their chemical bonding, which gives rise to nonclassical concepts of π/σ aromaticity, multiple aromaticity, antiaromaticity, and conflicting aromaticity. The unusual bonding pattern also leads to the dynamic structural fluxionality of bare boron clusters and relevant compound systems.

Doping or alloying with metal elements in boron clusters can effectively tune or alter the structures, electronic properties, and chemical bonding of boron-based alloy clusters. For cases of metal elements with low electronegativity as dopants (such as alkali metals or alkaline earth metals), boron-based alloy clusters should demonstrate intramolecular charge transfers from metal to boron, thus resulting in new types of boron structural motifs that are unknown in bare boron clusters. For transition metals as dopants, the situation appears to be more complicated. Boron-based alloy clusters include a rich variety of geometries: monocyclic boron rings, three-layered sandwiches, half-sandwiches, inverse sandwiches (or anti-sandwiches), tubes, drums, and heteroatomic molecular wheels [17,18,19,20,21]. Indeed, metal-doped boron-based binary clusters have recently emerged as a topic of interest in boron chemistry. In particular, inspired by the planar, circular boron molecular wheels and the high bonding capacity of d orbitals of transition metals, a series of metal-centered boron molecular wheel clusters, M^(*x*)^©B*_n_^k^*^−^, were designed and experimentally confirmed in recent years: Co©B_8_^−^, Ru©B_9_^−^, Ir©B_9_^−^, Ta©B_10_^−^, and Nb©B_10_^−^. These are structurally similar to bare boron wheel clusters, by substituting the central boron site with a transition metal atom [21,22,23,24]. On the basis of the electronic requirement for double π/σ aromaticity with (4*N*_π_ + 2) and (4*N*_σ_ + 2) electron counting, researchers proposed a design principle that the total number of bonding electrons (3*n* + *x* + *k*) [23] should be equal to 2*n* + 12. In other words, the electronic requirement for a central metal atom in anionic M^(*x*)^©B*_n_^k^*^−^ cluster is *x* = 12 − *n* − *k*, where *x* is the formal valence of a transition metal. Of course, it was assumed here that the metal center exhausts all valence electrons for chemical bonding in the cluster. For a binary system such as the title inverse sandwich cluster, this is not necessarily the case.

Although much progress has been made in the experimental or theoretical studies of transition-metal-doped boron clusters in recent years, there are relatively few works on boron alloy clusters doped with double transition metal atoms. By combining gas-phase PES experiments with quantum chemical calculations, Zhai et al. [25] studied the geometric structure and chemical bonding of a binary Au_2_B_7_^−^ cluster in 2006. Subsequently, the studies on Ta_2_B*_x_*^−^ (*x* = 2–5) clusters by Wang and coworkers and on the Sc_2_B*_n_* (*n* = 1–10) clusters by Jia et al. offered information on the competition between metal–metal, B–B, and metal–boron bonding in alloy boride clusters [26,27]. A joint PES experimental and quantum chemical work on Ta_2_B_6_^−/0^ clusters in 2014 showed that they assume a bipyramidal form, in which a B_6_ ring is sandwiched in between two Ta atoms [28], laying the groundwork for more complicated metal-doped M*_x_*B*_y_*^−^-type clusters. This structure may be alternatively described as an inverse sandwich. However, there was no discussion on direct Ta–Ta interaction in the cluster. In a more recent work, Wang and coworkers studied lanthanide-doped boron sandwich clusters, Ln_2_B*_n_* (*n* = 7, 9) [29]. A survey of the previous studies of double-transition-metal-doped boron clusters suggests that, except for a Ta_2_B_8_ cluster [30], it is rather difficult to form obvious metal–metal bonds between metal centers in inverse sandwich clusters.

Can the bimetal unit in boron-based inverse sandwich clusters act as a genuine dimer chemically, instead of two individual capping metal sites? As mentioned above, such examples are rare in the literature. If yes, is there an alternative way to elucidate this kind of novel cluster structure? What governs the structures of inverse sandwich clusters? Why can a monocyclic boron ring exist in inverse sandwich clusters, despite the fact that it is unstable in bare boron clusters? Is there a chemical connection between boron-based inverse sandwich alloy clusters and planar hypercoordinate molecular wheels? What are the peculiar aspects of chemical bonding in inverse sandwich clusters? All these are still open questions to date. In the present contribution, we attempt to address these issues using a properly chosen binary V_2_B_7_^−^ cluster as an example. Specifically, we have computationally designed an inverse sandwich cluster, *C*_s_ (^1^A’) V_2_B_7_^−^, through unbiased computer global-minimum (GM) searches and electronic structure calculations at the density-functional theory (DFT) levels. The inverse sandwich cluster is composed of a peripheral monocyclic B_7_ ring and a penetrating vertical V_2_ dimer. The latter structural unit is shown to possess a Lewis-type two-center two-electron (2c-2e) σ bond, which chemically represents, therefore, a real V_2_ dimer. Chemical bonding analysis indicates that the binary cluster is essentially held together by 26 valence electrons that constitute the skeleton σ bonds as well as delocalized 6π and 6σ frameworks. The inverse sandwich cluster shows double 6π/6σ aromaticity, conforming to the (4*n* + 2) Hückel rule. The overall bonding pattern closely mimics that of a bare B_8_^2−^ molecular wheel cluster. We propose that the V_2_ dimer in the binary V_2_B_7_^−^ cluster, which is formally in the [V_2_]^−^ charge state due to an extra charge in the anion, undergoes electronic transmutation [31,32], so that it becomes a single valence five site (analogous to B^2−^ or C^−^). This concept offers a natural chemical connection between inverse sandwich clusters and planar hypercoordinate molecular wheels. There are six extra electrons in binary V_2_B_7_^−^ cluster relative to bare B_8_^2−^ cluster [6], and they are consumed as a Lewis V–V σ bond and four nonbonding V d electrons (two for each V site, albeit not a lone pair). With this understanding, binary V_2_B_7_^−^ and bare B_8_^2−^ clusters should indeed be considered isoelectronic, which helps validate the introduction of the electronic transmutation concept into the present alloy cluster system.

## 2. Results

### 2.1. Global-Minimum Structure

Alternative optimized low-lying isomeric structures of the V_2_B_7_^−^ cluster system are shown in the Supplementary Materials (Figure S1) at the PBE0/def2-qzvp level, along with their relative energies (with corrections for zero-point energies, ZPEs). These low-lying structures are located in this work using the unbiased computer global searches (for both the singlets and triplets). Two additional sets of energetics data are also presented in Figure S1. Firstly, the PBE0-D3/def2-qzvp calculations are conducted for the top 15 structures, which serve to check the dispersion effect on energetics for the current system. Second, comparative B3LYP/def2-qzvp calculations, also for the top structures, are completed to check for computational consistency of different density functionals in terms of geometries and energetics. Note that, in the DFT methods, the PBE0 and B3LYP functionals are widely considered to be complementary to each other for a molecular system. As the results show, all three levels of theory generate highly coherent and consistent energetics data for the system (Figure S1). The GM cluster, *C*_s_ (^1^A’), is reasonably well-defined on the potential energy surface, which is at least ~0.6 eV below its nearest *C*_1_ (^3^A) competitor. This observation suggests that it is rather safe to assign the GM structure. A further benchmark of the energetics (such as at the single-point CCSD(T) level) would not offer much new information for the current system. In this article, we primarily discuss the computational data at the PBE0/def2-qzvp level.

**Figure 1:**
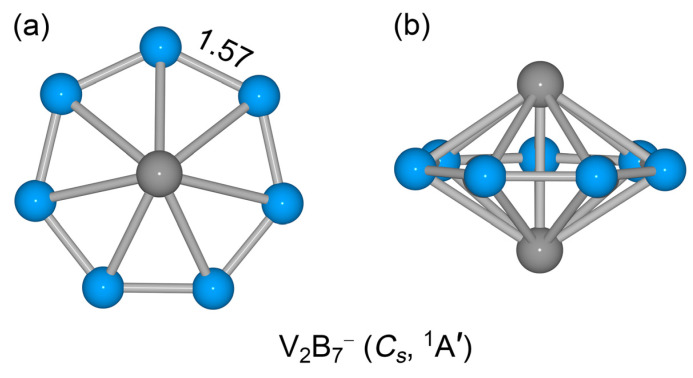
Optimized global-minimum (GM) structure of binary V_2_B_7_^−^ cluster at the PBE0/def2-qzvp level. Both (**a**) top and (**b**) side views are illustrated. Bond distances of peripheral B–B links (in Å) are also shown.

The top and side views of GM *C*_s_ (^1^A’) V_2_B_7_^−^ cluster are illustrated in Figure 1. It is a closed-shell electronic system. In the GM cluster, a monocyclic B_7_ ring is sandwiched between two capping V atoms, which may be classified as a new example of boron-based inverse sandwich clusters. The *C*_s_ GM structure appears to be moderately distorted from a higher *D*_7h_ symmetry. To address the issue of *C*_s_ versus *D*_7h_ geometries of the system, we have made further computational efforts.

The *D*_7h_ (^1^A_1_’) structure of V_2_B_7_^−^ cluster is confirmed not to be a true minimum at both the PBE0/def2-qzvp and B3LYP/def2-qzvp levels, which are complementary DFT methods as mentioned above. It is a second-order saddle point on the potential energy surface with two imaginary frequencies of 187.2*i* and 182.5*i* cm^−1^ at PBE0/def2-qzvp, as well as 152.3*i* and 146.1*i* cm^−1^ at B3LYP/def2-qzvp. Technically, the *D*_7h_ (^1^A_1_’) structure should be safely ruled out as a minimum for the present system. Nonetheless, the *D*_7h_ (^1^A_1_’) structure only lies 0.008 and 0.009 eV above *C*_s_ GM cluster at PBE0/def2-qzvp and B3LYP/def2-qzvp, respectively, which are far within the uncertainties of DFT methods. In other words, the *C*_s_ GM and *D*_7h_ structures are virtually isoenergetic with each other. We believe the distortion of *C*_s_ GM V_2_B_7_^−^ cluster is very likely a computational artifact of the DFT methods. Chemically, the *C*_s_ GM and *D*_7h_ structures of the system may be viewed as the same geometry. The optimized Cartesian coordinates for the GM *C*_s_ (^1^A’) and *D*_7h_ (^1^A_1_’) structures of V_2_B_7_^−^ cluster at PBE0/def2-qzvp are presented in Table S1.

### 2.2. Bond Distances, Wiberg Bond Indices, and Natural Atomic Charges

**Figure 2:**
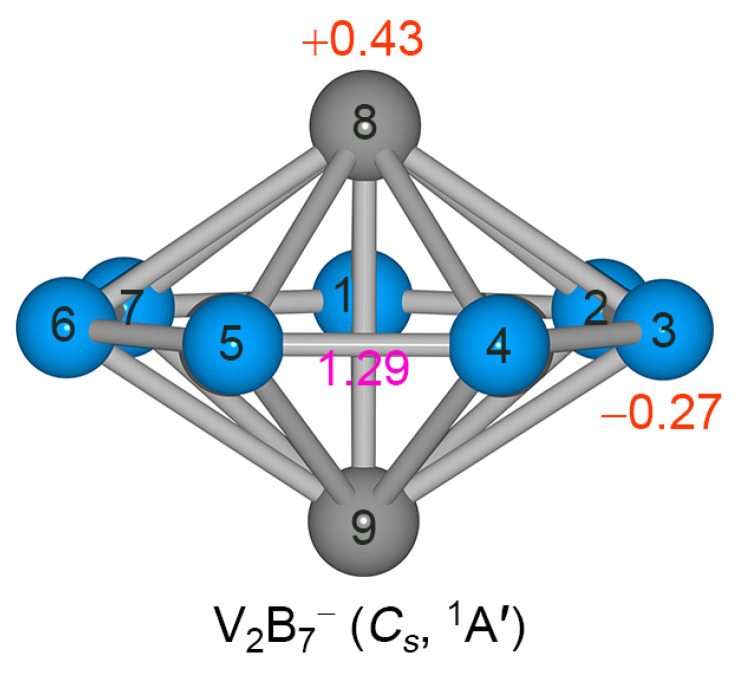
Calculated Wiberg bond indices (WBIs) of peripheral B−B links (in pink color) and natural atomic charges (in |e|, red color) for GM *C*_s_ (^1^A’) V_2_B_7_^−^ cluster. These values are obtained from natural bond orbital (NBO 6.0) analysis at the PBE0/def2-qzvp level.

The calculated bond distances, Wiberg bond indices (WBIs), and natural atomic charges of GM *C*_s_ (^1^A’) V_2_B_7_^−^ cluster are shown in Figure 1a and Figure 2, and Table S2; those of *D*_7h_ (^1^A_1_’) cluster are also presented in Table S2. The structural data offer key information for understanding a molecular system. According to the recommended covalent atomic radii [33], the upper bound of bond distance for a B–B, B=B, V–B, or V–V bond is approximately 1.70, 1.56, 2.19, and 2.68 Å, respectively. A typical B=B bond has a distance of 1.52 Å [34]. Based on these reference data, the peripheral B−B links (1.57 Å) in GM *C*_s_ (^1^A’) V_2_B_7_^−^ cluster are apparently shorter than a single bond, suggesting that these are beyond single B–B bond. In line with bond distances, their calculated WBI values (1.29; Figure 2) are clearly greater than 1.00. Thus, the peripheral B–B links are stronger than a single B−B bond, but weaker than a double bond, which is due to the fact that the B−B links have collective contributions from Lewis-type (or quasi-Lewis-type) 2c-2e B−B σ bonds as well as from globally delocalized π and σ frameworks (vide infra).

The V−B distances in GM *C*_s_ (^1^A’) V_2_B_7_^−^ cluster range from 2.13 to 2.18 Å (Table S2), which are relatively uniform despite its *C*_s_ distortion. The corresponding bond distances in the highly symmetric *D*_7h_ (^1^A_1_’) structure are 2.15 Å. In other words, the *C*_s_ distortion with respect to *D*_7h_ is less than 0.03 Å for V−B links, which is relatively minor. The calculated WBIs for V−B links in GM *C*_s_ (^1^A’) V_2_B_7_^−^ cluster are 0.49−0.40 (Table S2), which are substantial considering that each V center is coordinated to as many as seven B atoms and that the V–B bonding is of polar nature. The latter concept is understandable because the B and V elements possess quite different electronegativities: 2.04 versus 1.63. These WBI values demonstrate that the V–B interaction is dominated by covalent bonding. Most strikingly, the V−V distance in GM *C*_s_ (^1^A’) V_2_B_7_^−^ cluster is 2.34 Å, which is somewhat shorter than the upper bound of a single V−V bond (2.68 Å). This observation suggests that direct metal–metal bonding may be present in the inverse sandwich cluster, although a WBI bond order is known to be unreliable for such a bonding situation owing to the occurrence of an intermediate B_7_ ring in between two V atoms.

The calculated natural atomic charges from natural bond orbital (NBO) analysis [35] are shown in Figure 2. The B atoms each carry a slightly negative charge of –0.27 |e|. The V centers are each positively charged by +0.43 |e|. The net charge on a V center seems to be moderate in this anion cluster, again confirming that there is rather covalent V–B bonding. However, it should be noted that the polar nature of V–B bonding is more significant than the above numbers show, because in the anion cluster the extra charge is primarily located on the V_2_ unit (vide infra), which effectively compensates for the intramolecular charge transfers from V to B. The actual charge transfer associated with polar V–B bonding is estimated to be around 2 |e| in the system. This idea is strengthened using the comparative NBO data from a relevant neutral species, which is described below. In short, the V–B bonding in the alloy cluster, in both anionic and neutral charge states, is of mixed covalent and ionic nature.

## 3. Discussion

### 3.1. Chemical Bonding: CMO and AdNDP Analyses, ELF Analysis, and NICS Calculations

**Figure 3:**
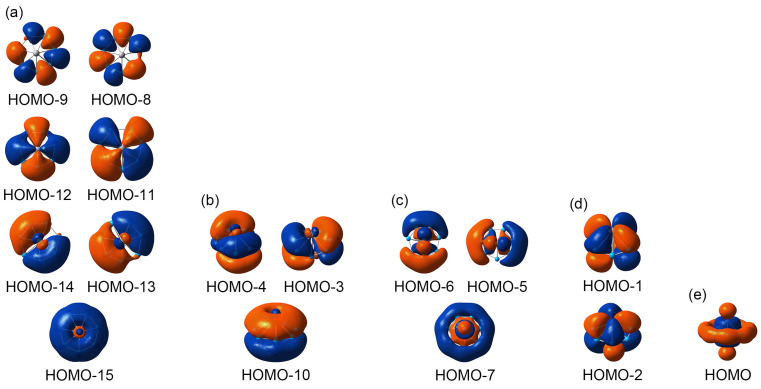
Pictures of canonical molecular orbitals (CMOs) of GM *C*_s_ (^1^A’) V_2_B_7_^−^ cluster. (**a**) Seven CMOs for peripheral four-center two-electron (4c-2e) V–B_2_–V σ bonds; see the text for detailed analysis. (**b**) Three delocalized π CMOs; that is, the π sextet. (**c**) Three delocalized σ CMOs. (**d**) Two nonbonding CMOs over two V atoms. (**e**) One 2c-2e V–V d_σ_ single bond.

In order to understand the unusual geometric structure of GM *C*_s_ (^1^A’) V_2_B_7_^−^ cluster, it is crucial to perform a thorough chemical bonding analysis. To this end, the canonical molecular orbital (CMO) analysis is of fundamental importance. The established bonding picture may be further confirmed or validated using adaptive natural density partitioning (AdNDP) analysis [36]. The GM *C*_s_ (^1^A’) V_2_B_7_^−^ cluster is closed-shell with 32 valence electrons. The 16 occupied CMOs are depicted in Figure 3, which can be classified into five subgroups according to their constituent atomic orbitals (AOs). Subgroup (a) contains seven CMOs. These are primarily composed of B 2s AOs, with secondary contribution from two V centers. They strictly follow the CMO building principles with 0, 1, 2, and 3 nodal planes from the bottom up, including three quasi-degenerate pairs. The seven CMOs (Figure 3a) can be recombined and localized as a set of Lewis-type or quasi-Lewis-type four-center two-electron (4c-2e) V−B_2_−V skeleton σ bonds, one for each peripheral B–B link. Here the localization as seven V−B_2_−V σ bonds is necessary because their CMOs involve a nonnegligible component from two V centers (vide infra). The skeleton σ framework consumes 14 valence electrons.

Subset (b) is the global π sextet of the cluster, with a spatial distribution that is closely similar to the prototypical π sextet in benzene. The subset is nine-centered in nature, with contributions from the V_2_ unit. The seven-fold symmetry of the inverse sandwich cluster suggests that the π sextet is essentially delocalized and cannot be localized as a Kekulé-type benzene. The 6π electron counting also conforms to the (4*n* + 2) Hückel rule for aromaticity. Thus, subset (b) renders π aromaticity to the inverse sandwich cluster. Likewise, subset (c) contains three σ CMOs, which are also global in nature. Their overall pattern mimics that of subset (b), except that the former CMOs are σ in nature. Again, these σ CMOs are essentially delocalized and cannot be reduced to Lewis-type σ bonds. It is imperative to claim σ aromaticity for the inverse sandwich cluster, which has 6σ electron counting that also conforms to the Hückel rule.

The remaining three CMOs in Figure 3, that is, subsets (d) and (e), are mainly based on the V_2_ unit and do not involve a major component of either B–B or V–B bonding. This statement lies in the fact that the B_7_ ring only represents a quite minor component in these CMOs. The two subsets are discussed in detail in Section 3.2. The above analysis indicates that chemical bonding in the inverse sandwich cluster is dictated by 26 valence electrons, of which 14 are responsible for skeleton σ bonds along the peripheral B_7_ ring (with a certain extent of 4c-2e V−B_2_−V expansion) and 12 are evenly split between the global 6π/6σ frameworks. The inverse sandwich cluster features double 6π/6σ aromaticity.

**Figure 4:**
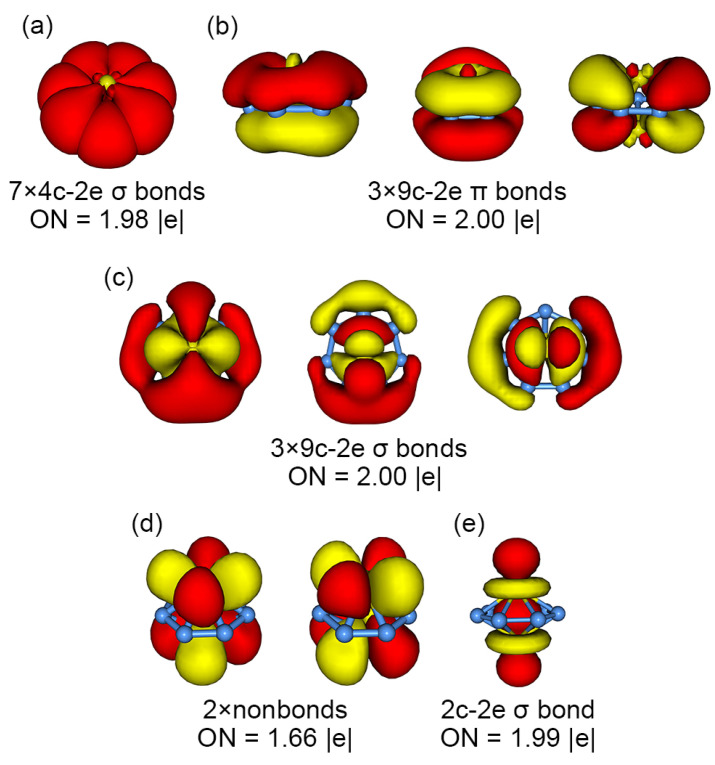
Chemical bonding scheme of GM *C*_s_ (^1^A’) V_2_B_7_^−^ cluster, according to adaptive natural density partitioning (AdNDP) analysis. Occupation numbers (ONs) are indicated. (**a**) Seven quasi-Lewis-type 4c-2e V–B_2_–V σ bonds along the periphery. (**b**) Global π sextet. (**c**) Global σ sextet. (**d**) Four nonbonding V 3d electrons, two for each V center (albeit not a lone pair). (**e**) One Lewis-type V–V d_σ_ single bond for metal–metal bonding in the system. An alternative AdNDP scheme is presented in Figure S2. The latter scheme is less than ideal, thus highlighting an intriguing difference between inverse sandwich clusters and planar molecular wheels, even in the skeleton σ bonding on the periphery.

This bonding picture is elegantly borne out from the AdNDP analysis. Our favorable AdNDP scheme is shown in Figure 4. The double 6π/6σ aromaticity in the cluster is well-produced in the AdNDP data (Figure 4b,c). The three V_2_-based bonding elements are also reproduced in the AdNDP data (Figure 4d,e). For skeleton σ bonding, the 4c-2e V−B_2_−V σ bonds give their occupation numbers (ONs) as 1.98 |e| (Figure 4a), which are nearly ideal values. The 4c-2e V−B_2_−V σ bonds are in line with the CMO component data. Our analysis shows that seven CMOs in subset (a) in Figure 3 collectively have a component of roughly ~1.7 |e| from the V_2_ unit.

Alternatively, the skeleton σ framework can be manually partitioned as seven Lewis-type 2c-2e B−B σ single bonds. Such an AdNDP scheme is shown in Figure S2. The latter AdNDP scheme has fundamental flaws. Firstly, the Lewis-type 2c-2e B−B σ single bonds have relatively low ON values of 1.81 |e| (Figure S2a), which are less than ideal. As a reference, a *D*_7h_ B_8_^2−^ cluster has ONs of 1.95 |e| for its peripheral 2c-2e σ single bonds. Second, as a result of the above artificial partitioning, the corresponding 6π and 6σ bonding elements become highly distorted relative to their CMOs; see Figure S2b,c. The latter observation is an indirect reflection that Lewis-type 2c-2e B−B σ single bonds are not the correct way of partitioning for the system. It is stressed that the roof-like 4c-2e skeleton σ bonds seem to be unique in an inverse sandwich cluster. The electron cloud manages to cover the entire surface of the cluster in a truly three-dimensional fashion, rather than the periphery only. A similar skeleton bonding feature is unknown in planar molecular wheel clusters.

As described above, characteristic double 6π/6σ aromaticity is firmly established in inverse sandwich GM *C*_s_ (^1^A’) V_2_B_7_^−^ cluster from the CMO and AdNDP analyses. To further assess double π/σ aromaticity in the system in a sort of quantitative manner, we calculate the nucleus-independent chemical shifts (NICSs) [37]. It is generally believed that NICS_zz_ is a simple and effective indicator of π/σ aromaticity of a molecular system. As shown in Figure S3, the NICS_zz_(0) values are calculated at the center of the inverse sandwich cluster (that is, the center of the B_7_ ring), as well as at the center of the quadrilateral B1B2B3B4 unit. The NICS_zz_(0) values are –70.1 and –74.3 ppm, respectively. In contrast, NICS_zz_(1) is calculated at 1 Å above the center of the quadrilateral B1B2B3B4 unit, which amounts to –63.4 ppm. Since NICS_zz_(1) and NICS_zz_(0) roughly probe π and σ aromaticity, respectively, the highly negative NICS_zz_(0)/NICS_zz_(1) values are a strong indication of π/σ aromaticity in the inverse sandwich cluster.

**Figure 5:**
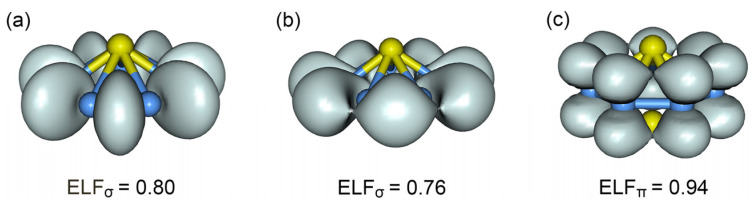
Electron localization functions (ELFs), ELF_σ_ and ELF_π_, for GM *C*_s_ V_2_B_7_^−^ cluster at the PBE0/def2-qzvp level. (**a**) ELF_σ_ associated with seven skeleton σ bonds. (**b**) ELF_σ_ associated with delocalized σ bonds. (**c**) ELF_π_ associated with delocalized π bonds.

The calculated electron localization functions (ELFs) [38,39] are also in line with double π/σ aromaticity in the cluster. The ELF_π_ and ELF_σ_ data are presented in Figure 5. Note that the bifurcation values for ELF_π_ (Figure 5c) and ELF_σ_ (Figure 5b) are 0.94 and 0.76, respectively, which are all greater than 0.7, thus indicating double π/σ aromaticity in the system.

### 3.2. On the Metal–Metal Bonding in Inverse Sandwich Cluster: Evidence for a V−V d_σ_ Bond

The nature of metal–metal bonding in boron-based inverse sandwich clusters has not been sufficiently elucidated in the literature. This issue is of crucial importance in order to achieve an in-depth understanding of chemical bonding in such systems. In Section 3.1, we only briefly mentioned that six of the valence electrons in GM *C*_s_ (^1^A’) V_2_B_7_^−^ cluster are primarily based on the V_2_ unit. The corresponding CMOs are depicted in Figure 3d,e, whereas their AdNDP bonding elements are shown in Figure 4d and 4e, respectively.

Subset (d) in Figure 3 has two CMOs that are mainly composed of V 3d AOs. These AOs are oriented parallel to the plane of B_7_ ring. Specifically, HOMO–1 is dominated by V 3d_xy_ AOs with a collective contribution of 81%, which is balanced by a secondary B 2p_z_ component. The CMO shows a destructive combination between two V centers, albeit with no direct overlap between their AOs. The coupling between two V 3d_xy_ AOs is only formal. The net bonding effect from this CMO is mediated by the middle B_7_ ring, with a minor B 2p_z_ component that interacts with both V centers in a constructive (or bonding) manner. Technically, each V center contributes ~0.8 |e| to this specific CMO. In a zeroth-order picture, this CMO can be approximated as two nonbonding V 3d electrons, one for each V center.

Likewise, HOMO−2 has an 82% component from V 3d_x_^2^_−y_^2^ AOs, also being balanced by a minor B 2p_z_ component. As a zeroth-order approximation, the CMO may be classified as two nonbonding V 3d electrons as well, one for each V center. The AdNDP scheme (Figure 4d) gives ON values of 1.66 |e|, which are partitioned as two-center nonbonding elements. These ONs are in line with the CMO component data. In short, subset (d) can be approximated as four nonbonding V 3d electrons (with secondary bonding effect as mediated by the B_7_ ring). Each V center in the inverse sandwich cluster has two nonbonding 3d electrons, although they do not represent a lone pair.

It is important to comment that HOMO–1 and HOMO−2 in Figure 3d should not be confused with the π sextet in Figure 3b. They are fundamentally different types of CMOs. Specifically, HOMO−4/HOMO−3/HOMO−10 in subset (b) of Figure 3 are dominated by B 2p_z_ AOs, with contributions that amount to 88.4%, 88.2%, and 74.5%, respectively. In stark contrast, HOMO–1 and HOMO−2 in subset (d) are primarily V-based as described above, with relatively minor contributions from B 2p_z_ AOs of 17.1% and 16.2%, respectively. These CMO component data differ distinctly. One should never consider 88.4%, 88.2%, 74.5%, 17.1%, and 16.2% as a single series of values. It would be conceptually incorrect to assign these five CMOs collectively as a 10π system, even though they seem to satisfy the (4*n* + 2) Hückel electron counting for π aromaticity. Such a claim of 10π aromaticity should not appear in the literature, for the sake of science.

For another technical comment, the relatively low ON values of 1.66 |e| in Figure 4d are natural, as long as one partitions them as 2c-2e nonbonding elements. The exact nature of their corresponding CMOs, that is, subset (d) in Figure 3, are fully described in the text. To consider these as 2c-2e nonbonding elements is only an approximation, as explicitly stated. In order to rationally evaluate how good such an approximation is, one may compare a two-center ON value of 1.66 |e| with an ideal nine-center ON value of 2.00 |e|. Note that the ratio of contribution from a B center and a V center herein is about 1:17. It is mentioned that AdNDP is a user-directed tool for bonding analysis, and the interpretation of its output requires expertise. One should not solely rely on the AdNDP data in order to understand a molecular system.

Last but not least, subset (e) in Figure 3 has one CMO, that is, the HOMO, which largely originates from V 3d_z_^2^ AOs from the V_2_ dimer with a collective contribution of 55.3%. In this CMO, the V 3d_z_^2^ AOs from two V sites are combined in a constructive, head-to-head manner with an apparent spatial overlap, owing to the short V–V distance (2.34 Å; see Section 2.2), which justifies direct V–V σ bonding in the inverse sandwich. The HOMO can be, therefore, roughly classified as a bonding CMO within the V_2_ dimer. To further assess metal–metal bonding in the system, a constructive/destructive recombination can technically be pursued between a pair of CMOs: HOMO and HOMO–7. Note that HOMO–7 also has a nonnegligible yet slightly smaller component of V 3d_z_^2^ AOs (by 42.4%). Thus, HOMO and HOMO–7 are indeed a pair of CMOs that are responsible for V–V bonding in the system. The constructive/destructive recombination of the pair is schematically illustrated in Figure S4.

**Figure 6:**
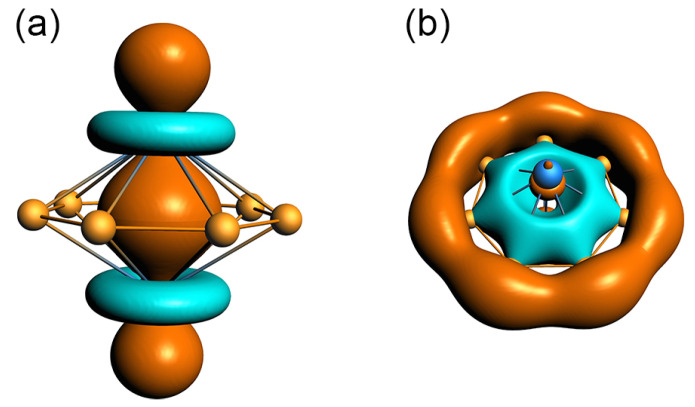
Two isolated and purified σ bonds associated with a pair of CMOs (HOMO and HOMO–7) in GM *C*_s_ V_2_B_7_^−^ cluster. (**a**) One Lewis-type 2c-2e V–V d_σ_ single bond. (**b**) One delocalized σ bond along the B_7_ ring. The latter is part of the aromatic σ sextet.

The outcome is presented in Figure 6. Here, HOMO–7 is “purified”, upon destructive recombination with HOMO, as a completely bonding CMO, which is strictly seven-centered in nature along the B_7_ ring (Figure 6b) and remains a component of the aromatic σ sextet. Likewise, upon constructive recombination with HOMO–7, HOMO is further strengthened for direct V–V σ bonding. This bonding element (Figure 6a) is purely two-centered in nature and solely situated on the V_2_ dimer, which has a spatial shape that shows straightforward evidence for bonding overlap between the V 3d_z_^2^ AOs from two V centers in a σ-bond manner. The ON value for this σ bond may be obtained from the AdNDP scheme, which is 1.99 |e| (Figure 4e), a perfect number. In short, our analysis clearly establishes an ideal, direct V–V σ single bond in inverse sandwich GM *C*_s_ (^1^A’) V_2_B_7_^−^ cluster. This result is remarkable and rather conclusive.

### 3.3. Electronic Transmutation: Analogy between an Inverse Sandwich Cluster and a Planar Molecular Wheel Cluster

The inverse sandwich GM *C*_s_ (^1^A’) V_2_B_7_^−^ cluster is quite unusual in terms of structure. It contains a monocyclic B_7_ ring and a perpendicularly penetrating V_2_ dimer. A monocyclic B_7_ ring is not present in bare boron clusters. A real V_2_ dimer with a Lewis-type V–V σ single bond is a rare example in inverse sandwich clusters. The seven-fold in-plane hypercoordination in the cluster suggests that the 6π/6σ frameworks are truly delocalized, thus rendering double π/σ aromaticity. To rationalize these peculiar aspects of the title inverse sandwich cluster, we would like to offer further discussion here. Our key idea is to introduce the concept of “electronic transmutation” [31,32] to the present system.

Specifically, the V_2_ unit in the system is, chemically rather than only physically, a genuine dimer. The chemical identity of the V_2_ dimer is established by a perfect Lewis-type σ single bond (Figure 6a). Thus, the V_2_ dimer should be capable of participating in chemical bonding as a single chemical unit, such as an individual atom. In terms of the number of valence electrons for the V_2_ dimer (Figure 4d,e), six electrons are consumed either as nonbonding electrons (two for each V site) or a Lewis σ single bond. The latter σ bond involves the contribution from an extra charge in the HOMO (Figure 3e and Figure 6a). In the context of bonding in an inverse sandwich cluster, a Lewis V–V σ single bond is relatively localized, which is therefore sort of equivalent to a lone pair in an atomic site. With this understanding, the V_2_ dimer, formally in its [V_2_]^−^ charge-state, is effectively valence five in terms of its capacity to participate in chemical bonding in the inverse sandwich system, because 6 out of its 11 electrons are actually “locked” or localized inside the dimer. In other words, the [V_2_]^−^ dimer in GM *C*_s_ (^1^A’) V_2_B_7_^−^ cluster is transformed to a single C^−^ or B^2−^ site via electronic transmutation. As a consequence, the GM V_2_B_7_^−^ cluster becomes chemically analogous to a *D*_7h_ CB_7_^−^ or B_8_^2−^ cluster [3,5,6,40]. Note that the formal charge state and the NBO charges should not be confused with each other; the latter are due to the polar nature of V–B bonding in the alloy system (see Section 2.2). In this way, a boron-based inverse sandwich cluster can be chemically equivalent to a planar molecular wheel system, which is an exotic chemical connection in the field.

**Figure 7:**
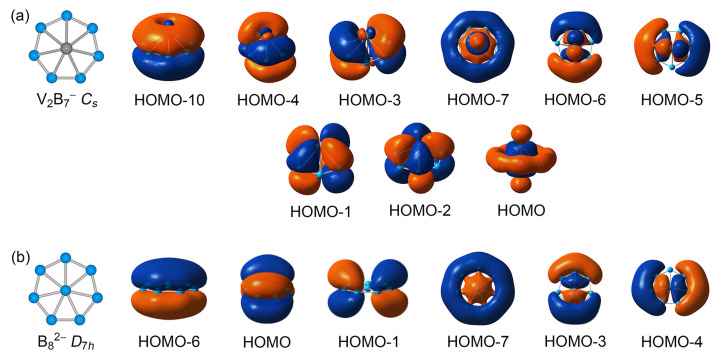
Comparison of delocalized π/σ CMOs of (**a**) GM *C*_s_ V_2_B_7_^−^ cluster with those of (**b**) a model *D*_7h_ B_8_^2−^ dianion cluster. The systems are not strictly isoelectronic to each other, because the former has six more valence electrons; see the three extra CMOs in (**a**). Nonetheless, these two systems are isovalent in terms of peripheral B–B σ bonds and delocalized 6π/6σ frameworks, which collectively consume a total of 26 electrons.

**Figure 8:**
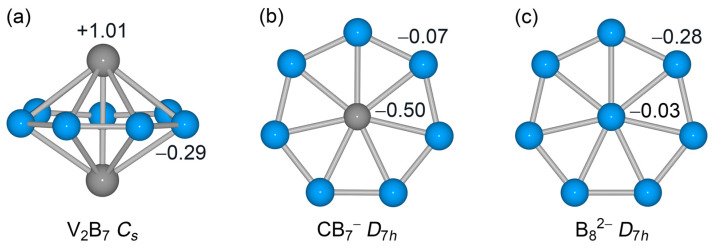
Calculated natural atomic charges (in |e|) for (**a**) neutral *C*_s_ V_2_B_7_, (**b**) model *D*_7h_ CB_7_^−^, and (**c**) model *D*_7h_ B_8_^2−^ clusters. The above data are obtained from natural bond orbital (NBO 6.0) analysis at the PBE0/def2-qzvp level.

A comparison between the present GM V_2_B_7_^−^ cluster and a model B_8_^2−^ cluster is presented in Figure 7. The delocalized π/σ CMOs of the two systems show close one-to-one correspondence with each other, as do their skeleton σ bonds (not shown). Note that HOMO−10, HOMO−5, HOMO−6, and HOMO−7 have components of 18.8%, 30.3%, 30.5%, and 42.4% V 3d AOs from the V_2_ dimer, respectively. Briefly, the five valence electrons from V_2_ dimer participate in three-fold bonding in the inverse sandwich. They contribute to skeleton σ bonding by approximately ~1.7 |e| and to delocalized 6σ framework by ~2.1 |e|, whereas their contribution to delocalized 6π framework is relatively minor (~0.4 |e|). This bonding pattern is identical to that of a model *D*_7h_ CB_7_^−^ cluster, in that the [V_2_]^−^ or C^−^ center is truly valence five (Figure 8b).

Nevertheless, GM *C*_s_ V_2_B_7_^−^ and *D*_7h_ B_8_^2−^ clusters show a subtle difference in the spatial distribution of their valence electrons. According to the natural atomic charges from NBO analysis [35], the two extra electrons in model B_8_^2−^ cluster are exclusively located on the B_7_ ring (Figure 8c), suggesting that it is best described as a neutral B center surrounded by a dianionic B_7_^2−^ ring, in which the hypercoordinate B center is actually valence three (rather than as B^2−^). Also shown in Figure 8 are the natural atomic charges in neutral *C*_s_ V_2_B_7_ cluster. A comparison between GM *C*_s_ V_2_B_7_^−^ (Figure 2) and *C*_s_ V_2_B_7_ (Figure 8a) clusters indicates that the extra charge in the anion is largely located on two V centers. This statement appears to be appropriate for at least two reasons. Firstly, the NBO charge on a V center changes from +1.01 |e| in the neutral to +0.43 |e| in the anion. In other words, upon addition of an extra electron, the V_2_ dimer gains over ~1.1 |e| (including electronic relaxation or redistribution, of course). Second, the HOMO of GM *C*_s_ V_2_B_7_^−^ cluster is mainly based on the V_2_ dimer (see Figure 3e, Figure 4e and Figure 6a), in which the extra electron in anion cluster occupies. Furthermore, the V–V distance in *C*_s_ V_2_B_7_ cluster is expanded to 2.54 Å (relative to 2.34 Å in GM V_2_B_7_^−^ cluster). This expansion is yet another piece of evidence for direct V–V σ bonding in GM *C*_s_ V_2_B_7_^−^ cluster. In neutral *C*_s_ V_2_B_7_ cluster, only five electrons are “locked” inside the V_2_ unit, because its HOMO is now only half-occupied. This observation further shows that the V_2_ unit is valence five in terms of bonding in the inverse sandwiches. It is reiterated that the net NBO charge of 1.01 |e| on a V center in *C*_s_ V_2_B_7_ cluster is due to the polar nature of V–B bonding. Therefore, an interesting implication from *C*_s_ V_2_B_7_ cluster is that the V–B bonding in inverse sandwich clusters do have a marked ionic component, despite bonding covalency.

It should be noted that the involvement of V_2_ dimer in three-fold chemical bonding in the inverse sandwich is a crucial factor that helps stabilize the binary system, which is also a demonstration of V–B bonding covalency in the cluster. While the contribution of electrons from a specific site may differ in V_2_B_7_^−^, CB_7_^−^, and B_8_^2−^ clusters, the three systems share the same 14 skeleton electrons, 6π aromaticity, and 6σ aromaticity. The systems only differ by three extra CMOs in V_2_B_7_^−^ cluster, as depicted in the second row in Figure 7a, which are primarily confined inside the V_2_ unit as nonbonding electrons or a Lewis-type V–V σ bond, as analyzed above. It is the general bonding pattern that chemically connects these systems, which validates the concept of electronic transmutation in the inverse sandwich cluster. We note that the present case is a fairly new type of electronic transmutation.

## 4. Methods Section

The GM structure of V_2_B_7_^−^ cluster was established in this work using the Coalescence Kick (CK) searches [41,42] at the PBE0/LANL2DZ level, which was also aided by manual structure constructions. The CK algorithm is believed to be capable of doing “mindless chemistry” [42], which is more than sufficiently powerful for the current simple alloy cluster system. A total of 3000 stationary points were probed on the singlet-state potential energy surface of the system, as were 3000 triplet-state geometries. The first 30 lowest-energy isomeric structures were then fully reoptimized at the PBE0/def2-qzvp level. Vibrational frequencies were also analyzed at the same level to ensure that the reported structures are true minima on the potential energy surface. The relative energies of all isomers presented in this paper were corrected for the ZPEs.

In order to check for computational consistency of the DFT methods in geometries and energetics, we made further efforts for the current cluster system. Firstly, the top 15 low-lying isomers were recalculated at the PBE0-D3/def2-qzvp level, which served to examine the dispersion effect. Such an effect turned out to be insignificant for the present system. Second, different density functionals were checked using independent and comparative computational data at the B3LYP/def2-qzvp level, also for the first 15 alternative structures. It is stressed that the PBE0 and B3LYP approaches are generally considered to be complementary to each other in quantum chemistry. The above extra efforts also included vibrational frequency analyses as well as ZPE corrections for energetics. Thus, the three levels of theory should collectively lead to rather solid conclusions for the present system. All three levels of theory reached the same GM structure for V_2_B_7_^−^ cluster. Since the three sets of computational data are highly coherent, we primarily focus on the PBE0 data in this paper.

The WBIs and natural atomic charges were calculated using the NBO analysis (NBO 6.0) [35,43], which was completed at the PBE0/def2-qzvp level. Chemical bonding was elucidated using CMO analysis, as well as AdNDP [36] and ELF analyses [38,39]. To independently evaluate aromaticity, NICSs [37] were calculated at PBE0/def2-qzvp. The CMO compositions were analyzed using the Multiwfn program [44]. The AdNDP data were visualized using Molekel [45]. All the electronic structure calculations were accomplished using the Gaussian 09 program [46].

## 5. Conclusions

We have computationally designed a new example of boron-based inverse sandwich clusters, *C*_s_ (^1^A’) V_2_B_7_^−^, through unbiased global-minimum (GM) structure searches and quantum chemical calculations. It is close to a highly symmetric *D*_7h_ system. The GM cluster features a monocyclic B_7_ ring on the periphery and a perpendicularly penetrating V_2_ dimer. The V sites have seven-fold hypercoordination with boron in a quasi-planar fashion. The inverse sandwich cluster is governed by double 6π/6σ aromaticity, following the (4*n* + 2) Hückel rule. The skeleton σ bonding in the system involves a marked contribution from the V_2_ dimer, which should be best described as a set of roof-like, four-center two-electron (4c-2e) V–B_2_–V σ bonds, rather than strict Lewis-type 2c-2e B–B σ single bonds. Interesting, the V_2_ dimer possesses a genuine 2c-2e V–V σ bond and represents a rare case of direct metal–metal bonding in inverse sandwich clusters. Despite its 11 valence electrons, the V_2_ dimer in its formal [V_2_]^−^ charge state in the alloy cluster is chemically analogous to a single atom with valence five, such as a C^−^ or B^2−^ site. This is a new type of “electronic transmutation”. The remaining six electrons in the V_2_ dimer are merely present as spectators in terms of chemical bonding within the inverse sandwich, including four nonbonding electrons and a Lewis-type V–V σ bond. Note that in the corresponding neutral inverse sandwich *C*_s_ V_2_B_7_ cluster, the V_2_ unit is also effectively valence five in terms of global chemical bonding. The anionic and neutral clusters only differ in their V–V σ bonding; that is, a σ single bond versus a σ half bond. The above understanding offers a natural connection between an inverse sandwich cluster and a planar hypercoordinate molecular wheel, such as that between V_2_B_7_^−^, CB_7_^−^, and B_8_^2−^ clusters. It is believed that the concept of electronic transmutation may be further developed to elucidate novel molecular structures and gain new physical chemistry.

## Data Availability

The data from this work are available in the manuscript as well as in the electronic Supplementary Materials file.

## Supplementary Materials

The following supporting information can be downloaded at: https://www.mdpi.com/article/10.3390/molecules28124721/s1, Table S1: Cartesian coordinates for GM *C*_s_ (^1^A’) and *D*_7h_ (^1^A_1_’) structures of V_2_B_7_^−^ cluster at the PBE0/def2-qzvp level; Table S2: Calculated bond distances and Wiberg bond indices (WBIs) of GM *C*_s_ (^1^A’) and *D*_7h_ (^1^A_1_’) structures at PBE0/def2-qzvp; Figure S1: Alternative optimized low-lying isomeric structures of V_2_B_7_^−^ cluster at PBE0/def2-qzvp, along with their relative energies at three levels of theory; Figure S2: An alternative bonding scheme of GM V_2_B_7_^−^ cluster based on AdNDP analysis; Figure S3: NICS_zz_ data for GM V_2_B_7_^−^ cluster at PBE0/6-311+G*; Figure S4: Schematic presentation of constructive/destructive recombination between HOMO and HOMO–7 of GM V_2_B_7_^−^ cluster that generates a “purified” Lewis-type V–V σ single bond.
