# Supplementary material for: Boron-Based Inverse Sandwich V2B7− Cluster: Double π/σ Aromaticity, Metal–Metal Bonding, and Chemical Analogy to Planar Hypercoordinate Molecular Wheels
Source: Molecules. 2023 Jun 12;28(12):4721. doi: 10.3390/molecules28124721 (PMC10305064; doi:10.3390/molecules28124721)
Supplement: Supplementary file 1 [file molecules-28-04721-s001.zip › molecules-2413516-supplementary.pdf]

molecules

Supplementary Materials

Boron-Based Inverse Sandwich $V_2B_7^-$ Cluster: Double π/σ Aromaticity, Metal-Metal Bonding, and Chemical Analogy to Planar Hypercoordinate Molecular Wheels

Peng-Fei Han [1], Qiang Sun [2] and Hua-Jin Zhai [1,*]

[1] Nanocluster Laboratory, Institute of Molecular Science, Shanxi University, Taiyuan 030006, China; 201722801006@email.sxu.edu.cn (P.-F.H.)
[2] Center for Applied Physics and Technology, School of Materials Science and Engineering, Peking University, Beijing 100871, China; sunqiang@pku.edu.cn
* Correspondence: hj.zhai@sxu.edu.cn

Table S1. Cartesian coordinates for the global-minimum (GM) structure, C_s ($^1A'$), of $V_2B_7^-$ cluster at the PBE0/def2-qzvp level. Also shown are those for a high symmetry D_{7h} ($^1A_1'$) structure, which is a second-order saddle point on the potential energy surface.

(a) GM, $V_2B_7^-$ (C_s, $^1A'$)

B	-0.01043500	-1.80676600	0.00000000
B	0.03795400	0.40036800	1.76112500
B	-0.07018600	-1.12503600	-1.41170800
B	0.03795400	0.40036800	-1.76112500
B	0.03795400	1.62708500	0.78405300
B	0.03795400	1.62708500	-0.78405300
B	-0.07018600	-1.12503600	1.41170800
V	1.17112700	-0.03378500	0.00000000
V	-1.17134700	0.03420500	0.00000000

(b) $V_2B_7^-$ (D_{7h}, $^1A_1'$)

B	0.00000000	1.80563900	0.00000000
B	1.76036800	-0.40179200	0.00000000
B	-1.41170500	1.12579800	0.00000000
B	-1.76036800	-0.40179200	0.00000000
B	0.78343700	-1.62682500	0.00000000
B	-0.78343700	-1.62682500	0.00000000
B	1.41170500	1.12579800	0.00000000
V	0.00000000	0.00000000	1.17125300
V	0.00000000	0.00000000	-1.17125300

Table S2. Calculated bond distances (BD; in Å) and Wiberg bond indices (WBIs) of GM $V_2B_7^-$ cluster at the PBE0/def2-qzvp level. The relevant computational data of D_{7h} $V_2B_7^-$ structure (second-order saddle point) are also shown as a footnote in the table.

Link[a,b]	V8–B1	V8–B2	V8–B3	V8–B4	V8–B5	V8–B6	V8–B7
BD	2.14	2.18	2.13	2.16	2.14	2.16	2.16
WBI[c]	0.47	0.40	0.49	0.43	0.47	0.43	0.43
Link	V9–B1	V9–B2	V9–B3	V9–B4	V9–B5	V9–B6	V9–B7
BD	2.17	2.13	2.18	2.13	2.17	2.15	2.15
WBI[c]	0.42	0.49	0.40	0.49	0.42	0.45	0.45

[a] Cluster structure and atomic labels are shown in Figure 2.
[b] Calculated BDs of D_{7h} $V_2B_7^-$ structure are 1.57 Å for the B–B links and 2.15 Å for V–B links. Their corresponding WBI values are 1.29 and 0.45, respectively.
[c] The WBIs are obtained using the NBO 6.0 program.

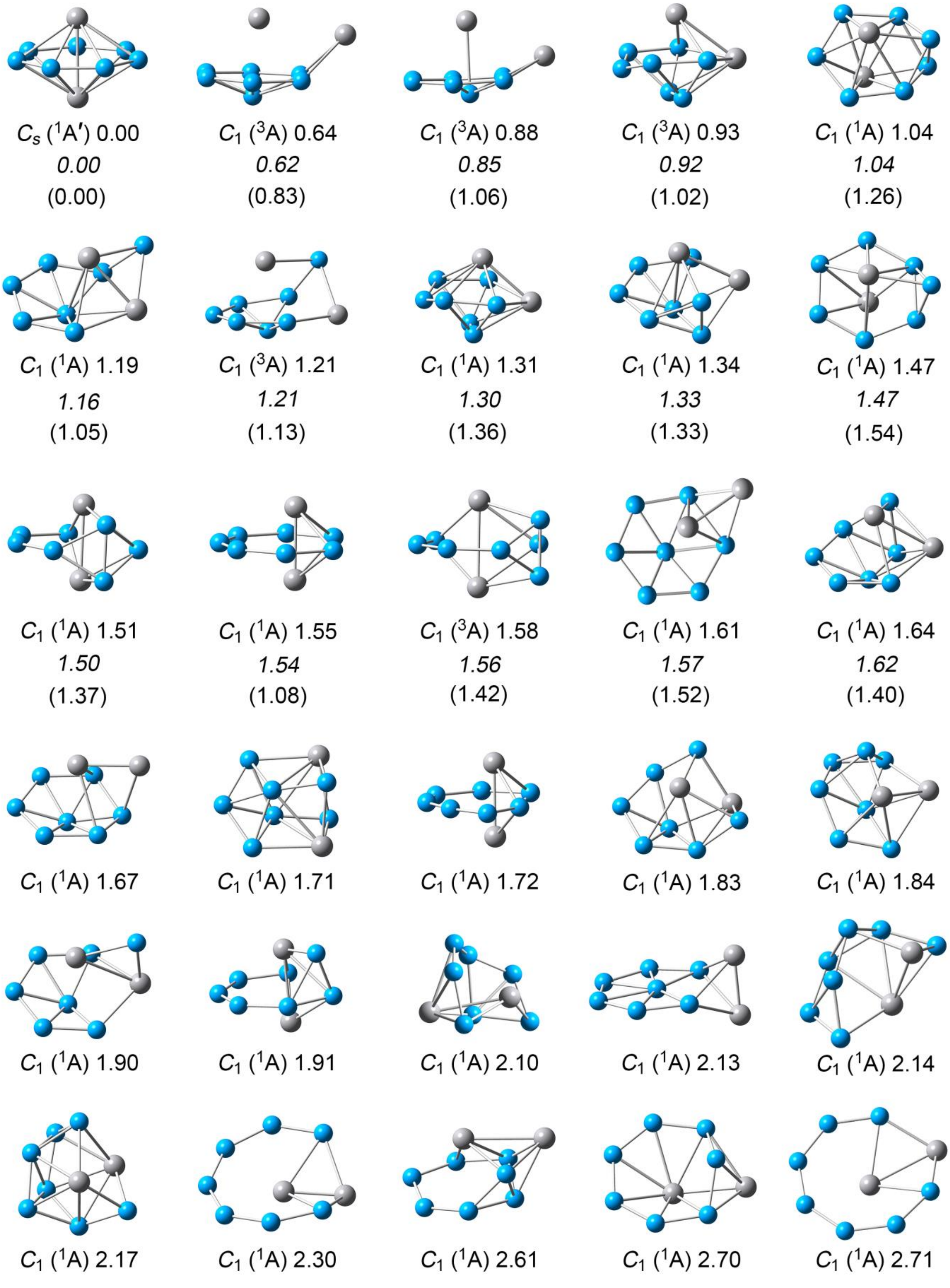

Figure S1. Alternative optimized low-lying isomeric structures of $V_2B_7^-$ cluster at the PBE0/def2-qzvp level along with their relative energies (shown in the first row), including zero-point energy (ZPE) corrections. The energetics data are also presented for the top fifteen structures at the PBE0-D3/def2-qzvp (the second row, in italics) and B3LYP/def2-qzvp (the third row, in parentheses) levels. All energies are shown in eV. The three sets of energetics data appear to be coherent with each other.

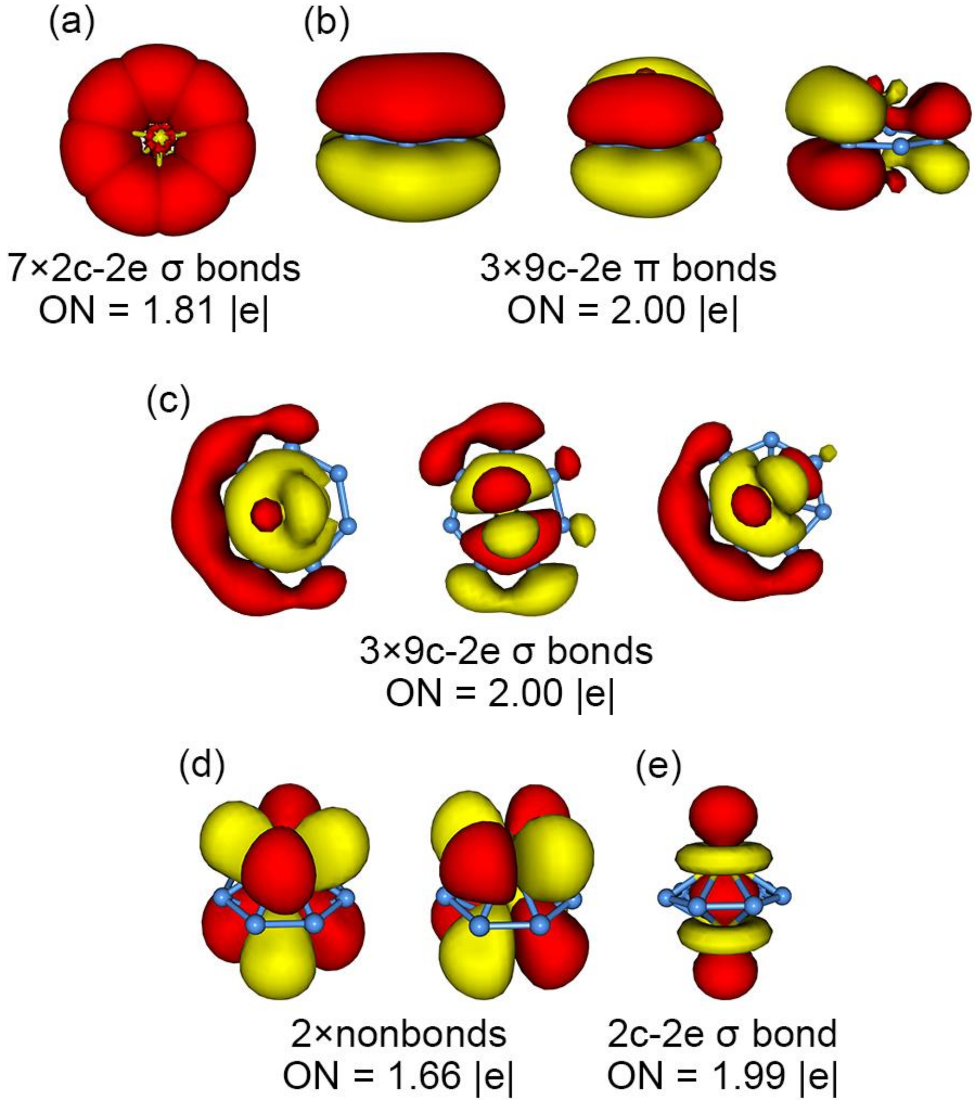

Figure S2. An alternative bonding scheme of GM C_s ($^1A'$) $V_2B_7^-$ cluster on the basis of adaptive natural density partitioning (AdNDP) analysis. Occupation numbers (ONs) are indicated. The AdNDP scheme differs from that in Figure 4 primarily in the peripheral B–B σ bonding, which is treated here strictly as Lewis-type two-center two-electron (2c-2e) σ bonds, thus leading to ONs (1.81 |e|) that are relatively less than ideal. Consequently, additional delocalized bonding elements in (b) and (c) are distorted as compared to the corresponding canonical molecular orbitals (CMOs), despite their ideal ON values of 2.00 |e|.

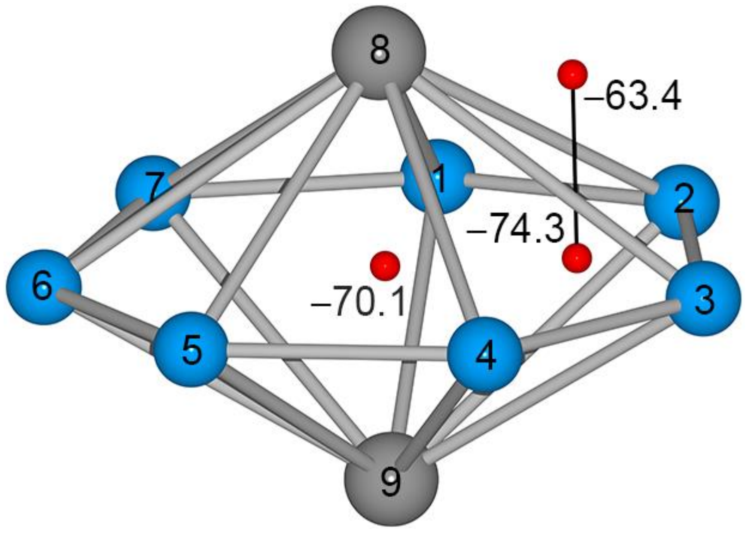

Figure S3. Calculated NICS data for GM $V_2B_7^-$ cluster at the PBE0/6-311+G* level. The $NICS_{zz}(0)$ values are obtained at the center of the B_7 plane and at the center of quadrilateral B1B2B3B4 unit. The $NICS_{zz}(1)$ value is obtained at 1 Å above the center of quadrilateral B1B2B3B4 unit.

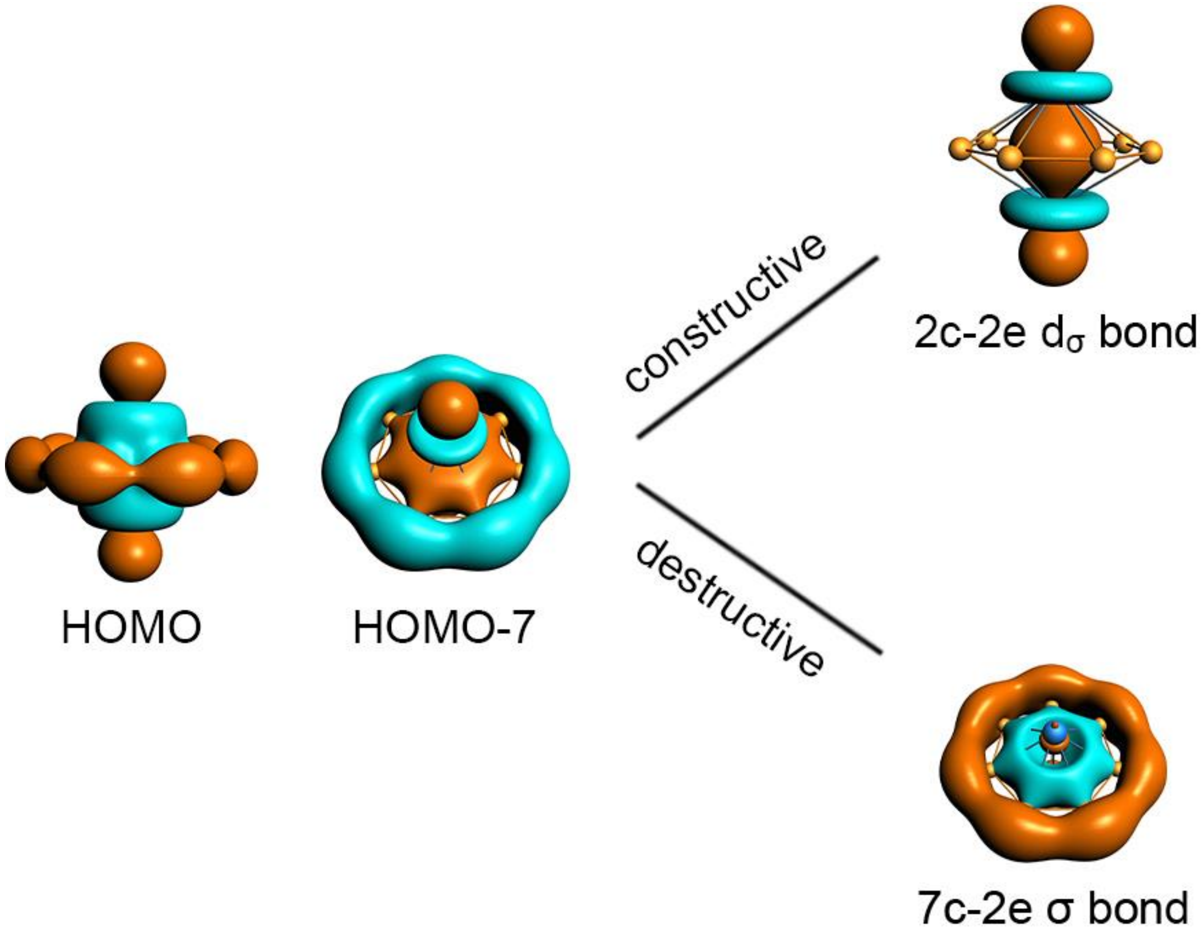

Figure S4. A schematic presentation of the constructive/destructive recombination between HOMO and HOMO–7 of GM $V_2B_7^-$ cluster. This treatment generates two relatively isolated and purified σ bonds, one for Lewis-type V–V σ bond and the other for delocalized σ bonding along the B_7 ring.
